# Cross-Sectional Investigations on Epitaxial Silicon Solar Cells by Kelvin and Conducting Probe Atomic Force Microscopy: Effect of Illumination

**DOI:** 10.1186/s11671-016-1268-1

**Published:** 2016-02-01

**Authors:** Paul Narchi, Jose Alvarez, Pascal Chrétien, Gennaro Picardi, Romain Cariou, Martin Foldyna, Patricia Prod’homme, Jean-Paul Kleider, Pere Roca i Cabarrocas

**Affiliations:** TOTAL New Energies, 24 cours Michelet, 92069 Paris, La Défense Cedex France; LPICM, CNRS, Ecole Polytechnique, Université Paris-Saclay, 91128 Palaiseau, Cedex France; IPVF (Institut Photovoltaïque d’Ile-de-France) - 8, Rue de la Renaissance, F-92160 Antony, France; GeePs, UMR CNRS 8507, CentraleSupelec, Univ Paris-Sud, Sorbonne Universités, UPMC Univ Paris 06, 11 rue Joliot Curie, Plateau de Moulon, 91192 Gif sur Yvette, France

**Keywords:** Epitaxial silicon, Solar cell, Kelvin probe force microscopy, Conducting probe atomic force microscopy, Photovoltage, Photocurrent

## Abstract

Both surface photovoltage and photocurrent enable to assess the effect of visible light illumination on the electrical behavior of a solar cell. We report on photovoltage and photocurrent measurements with nanometer scale resolution performed on the cross section of an epitaxial crystalline silicon solar cell, using respectively Kelvin probe force microscopy and conducting probe atomic force microscopy. Even though two different setups are used, the scans were performed on locations within 100-μm distance in order to compare data from the same area and provide a consistent interpretation. In both measurements, modifications under illumination are observed in accordance with the theory of PIN junctions. Moreover, an unintentional doping during the deposition of the epitaxial silicon intrinsic layer in the solar cell is suggested from the comparison between photovoltage and photocurrent measurements.

## Background

Since the invention of atomic force microscopy (AFM) at the end of the 1980s [1], various AFM extensions have been developed to perform a wide range of measurements at the nanoscale [2]. For instance, the most advanced electrical AFM extensions show the ability to explore simultaneously local electrical, mechanical, and surface topography properties. Conducting probe AFM (CP-AFM) and Kelvin probe force microscopy (KPFM) enable, respectively, to sense the local sample current and surface potential and correlate them with the surface morphology. Both techniques have gained importance as the new developments in the microelectronic industry often imply nanostructures which require micro/nanoscale analysis for a better understanding of electrical failures [3, 4].

The same AFM electrical extensions have also gained interest in the photovoltaic community. In particular, they have proven to be powerful tools for solar cell junction analysis. Indeed, the high spatial resolution of these techniques enables detailed cross section analysis of solar cells under various operating conditions, provided by an external illumination and voltage bias. In contrast with scanning electron and optical microscopy techniques which currently work in the high injection regime, these AFM extensions can perform measurements under real operating injection conditions. Therefore, they are unique tools to monitor the effect of illumination and electrical bias on solar cell devices at the nanoscale.

CP-AFM techniques have, for instance, been used to analyze the photocurrent of solar cells and photodetectors [5, 6], and in particular, the most advanced characterizations were performed on the cross section of solar cells evidencing the presence of inversion layers [7] and the influence of light on local resistivity [8]. Photovoltage measurements on the cross section using KPFM techniques have also been conducted on various types of solar cells such as III–V multi-junctions [9] or CuIn_*x*_Ga_(1 − *x*)_Se_2_ (CIGS) heterojunctions [10] revealing photogeneration effects and local surface potential shifts with the illumination intensity. The influence of the wavelength of illuminating light has also been reported [11]. More recently, it has been shown that nanoscale photovoltage measurements using a pulsed light source enable to extract the photocarrier lifetime [12].

In this paper, we compare photocurrent and photovoltage measurements performed within a distance of only 100 μm from each other, on the cross section of a crystalline silicon solar cell. The geolocation was made possible by the area indented after photocurrent measurements. We chose to use an epitaxial silicon solar cell fabricated by low-temperature plasma-enhanced chemical vapor deposition (PECVD) [13–15]. Our results show that both techniques provide results consistent with the expected solar cell behavior under illumination. Besides, a new way of performing photocurrent measurements is introduced facilitating the interpretation of the results. Measurement artifacts and reproducibility issues are also discussed. The cross analysis of photovoltage and photocurrent lead us to raise a hypothesis on the unintentional doping of the epitaxial silicon layer and the presence of a defective layer at the interface between the epitaxial layer and the crystalline silicon wafer.

## Methods

### Epitaxial Silicon Solar Cell

Recently, epitaxial silicon solar cells have gained attraction from the photovoltaic industry since they require less silicon material than traditional crystalline silicon solar cells, while reaching competitive efficiencies. For instance, Solexel achieved 21.2 % efficiency using 35-μm wafers [16]. Epitaxial silicon solar cells using plasma-enhanced chemical vapor deposition (epi-PECVD) have been developed in our laboratory (LPICM) enabling epitaxy at very low temperatures, below 200 °C [13–15].

Heavily boron-doped (100)-Si wafers with a resistivity of 0.002–0.005 Ω.cm and a thickness of 525 μm were used as substrates for the epitaxial growth. Non-intentionally doped (intrinsic) epitaxial layers with a thickness of 3 μm were deposited in a radio frequency plasma-enhanced chemical vapor deposition (RF-PECVD) reactor from the dissociation of 6 % silane in a hydrogen gas mixture (SiH_4_/(SiH_4_ + H_2_) = 0.06) under a pressure of 2 Torr and an RF power density 50 mW.cm^−2^, resulting in a deposition rate of 0.15 nm.s^−1^. An n^++^ hydrogenated amorphous silicon (a-Si:H) layer with a thickness of 15 nm is deposited on the epi-Si surface without breaking the vacuum at a constant temperature of 175 °C. The area of the cell (2 × 2 cm^2^) was defined by sputtering indium tin oxide (ITO) through a shadow mask and evaporating an aluminum contact grid above.

**Fig. 1:**
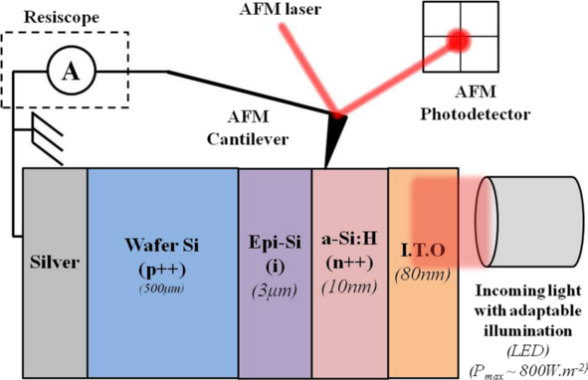
Schematic of the setup for photocurrent measurements on the thin epitaxial crystalline silicon solar cell

To perform electrical measurements on the cross section, the solar cell was cleaved and the cross section was polished mechanically using diamond grinding disks to achieve a corrugation less than 50 nm. A low surface roughness is necessary to perform electrical scanning probe microscopy measurements because topographical inhomogeneities can induce a topographical image imprint on the electrical image. Finally, the solar cell is placed on a sample holder which incorporates electrical contacts and an optical fiber to perform measurements under illumination. A light-emitting diode (LED) with a wavelength of 625 nm is injected in the optical fiber, and a LED driver enables to vary the intensity of the light (Fig. 1). The solar cell mounted in the AFM setup is grounded through the highly doped crystalline wafer and characterized under open circuit conditions.

### Conducting Probe AFM

Resiscope is an extension of the conducting probe AFM. It enables nanoscale current measurement using a conductive AFM probe in contact mode in the range between 10^-12^ A and 10^-2^ A thanks to a logarithmic current amplifier [17]. In our setup, Resiscope measurements are performed in ambient air using a Digital Instruments Nanoscope IIIa Multimode AFM. The probes used were silicon probes coated with boron-doped diamond. The stiffness of the cantilever was 48 N/m, and from the force/distance spectroscopy, we have determined that the force applied by the tip on the sample during measurements was around 10 μN. This force is one order of magnitude higher than the force applied during photocurrent SSRM measurements by Li et al [8].

Contrary to previous works on photoconductive AFM measurements [6, 8], we adapted our Resiscope setup to be able to perform measurements with no voltage bias applied externally between the tip and the sample (Fig. 1). Therefore, the only current source in the electrical circuit is the solar cell itself when illuminated. This configuration makes the interpretation of results much easier.

As we increase the light intensity, the photocurrent increases in the epitaxial silicon and a-Si:H layer but no photocurrent appears in the crystalline wafer since it is grounded and highly doped. The photocurrent increase in the epitaxial layer can be understood as a decrease of the contact resistance. Werber et al. explained that the measured contact resistance *R*_c_ is the sum of a geometrical resistance *R*_g_ and a barrier resistance *R*_b_ [18]. For a low contact radius *a* between the sample and the tip, *R*_c_ is very close to *R*_b_ and decreases exponentially with the electron concentration *n*. With increasing illumination, the electron concentration in the epitaxial layer increases. Hence, *R*_c_ which is predominant in the circuit, decreases exponentially. Therefore, we expect an exponential photocurrent increase with illumination.

###  Kelvin Probe Force Microscopy

Kelvin probe force microscopy [1] measures the contact potential difference (CPD) which is equal to the difference between the sample work function (WF_sample_) and the AFM tip work function (WF_tip_). Since WF_tip_ remains the same during the scans, KPFM images provide the evolution of WF_sample_ also called surface potential.

In our work, the solar cell is in open circuit conditions (Fig. 1). Under illumination, the quasi-Fermi levels of holes and electrons split. This affects the surface potential profile. The difference between surface potential with and without light is called surface photovoltage. The evolution of the photovoltage along the PIN junction is expected to change as follows:

- On the p^++^ doped crystalline silicon wafer, the surface potential remains unchanged under illumination because the wafer is highly doped and grounded. The quasi-Fermi level of holes under illumination is the same than the Fermi level under dark conditions. The photovoltage is equal to 0 V on the wafer.
- On the n^++^ a-Si:H layer, surface potential illumination is defined by the difference between the vacuum level and the quasi-Fermi level of electrons because electrons are majority carriers in this layer. The vacuum level remains constant with increasing light intensity. Under illumination, the quasi-Fermi level of electrons in the n^++^ a-Si:H layer splits from the quasi-Fermi level of holes in the p^++^ c-Si wafer by the open circuit voltage (*V*_oc_) value if we assume that surface effects are negligible. This is explained in detail in the theory of photovoltage on pn junction [19]. Therefore, the surface potential on the n^++^ a-Si:H layer is supposed to decrease by a value close to the *V*_oc_ with increasing illumination.
- On the intrinsic epitaxial layer, the photovoltage profile decreases progressively from 0 V on the p^++^ doped crystalline silicon wafer to the *V*_oc_ on the n^++^ doped a-Si:H layer.

We used an Agilent 5500 setup to perform KPFM measurements in amplitude modulation (AM) mode. Silicon probes with Pt/Ir coating and a force constant of 2.7 N/m were used at a resonance frequency around 60 kHz. With this setup, the surface potential measurements were recorded at the same time as topography (single pass mode). Since the tip is very close to the surface, it has the advantage of having small averaging effects due to stray capacitance. The measurements were performed in nitrogen atmosphere to minimize the effects of tip induced oxidation.

The surface potential or surface work function measured by KPFM corresponds to the local difference between the vacuum energy level and the Fermi energy level, underneath the tip.

### Measurements Timeline

The photocurrent measurements using CP-AFM were performed first, and the photovoltage measurements using KPFM were performed afterwards. At each step, macroscopic current-voltage (*I*-*V*) characteristics of the 0.5 cm^2^ solar cell were measured to make sure that the diode did not degrade during measurements. A small increase on series resistance of 5 % was observed after successive measurements but it is not detrimental to the interpretations because the *V*_oc_ induced by the maximum illumination intensity remains almost the same at each step of the measurement timeline.

In order to perform photovoltage and photocurrent measurements in the same conditions on the two AFM setups, we did not change the position of the cell on the cross section holder during the whole measurement campaign. Besides, the two measurements were performed within 100 μm distance. The positioning was possible because the area of scan where the photocurrent was first measured could be clearly identified as an indentation made by the tip near the edge.

**Fig. 2:**
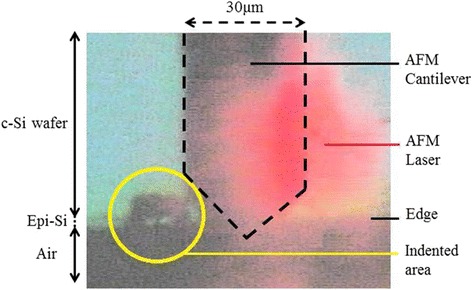
Top view image provided by the AFM camera. One can clearly see the position of the cantilever next to the indented area before performing photovoltage measurements

Figure 2 shows the image of the cantilever position next to the indented area (as detected by the KPFM camera) during the photovoltage measurements. Since the width of the cantilever is 30 μm, the distance between CP-AFM and KPFM areas of measurement can be estimated to be less than 100 μm. However, the distance between the two areas is large enough to avoid a detrimental effect of the indented area on photovoltage measurements. It can be noted that the KPFM measurements did not cause a visible degradation of the scanned area, as opposed to the CP-AFM measurements. This is because KPFM uses light tapping AFM mode whereas CP-AFM uses here hard contact AFM mode.

## Results and Discussion

### Influence of Illumination on KPFM and CP-AFM Images

**Fig. 3:**
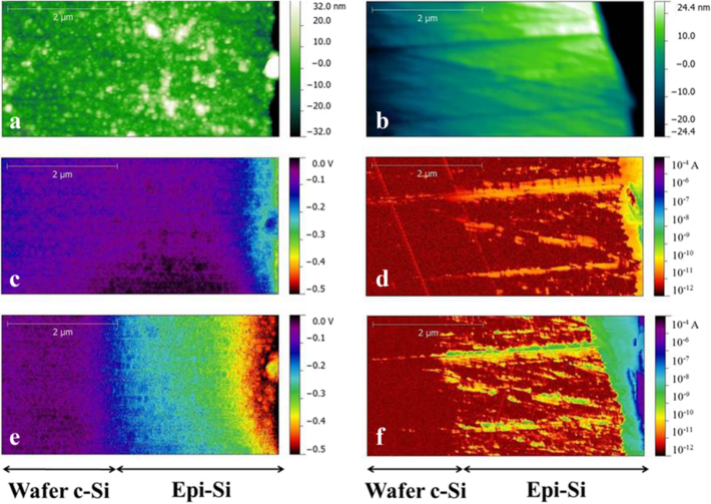
Topography image associated respectively to surface potential (KPFM) and current (CPAFM) measurements, **a** and **b**, respectively. Surface potential and current images without LED, **c** and **d**, respectively and at full LED illumination, **e** and **f**, respectively. The scan area is 2.5 × 5 μm for all the images

Figure 3 shows KPFM and CP-AFM images with and without LED illumination and the topography images associated to each of them. Full illumination corresponds to a light intensity of 800 W/m^2^ which is close to one sun (1000 W/m^2^) since the open circuit voltage is 460 mV under full LED illumination, comparable to 530 mV measured in a solar cell simulator under one sun. Scanning dimensions are 2.5 × 5 μm. The edge of the sample with the n^++^ a-Si:H layer is on the right side of the picture. It can be noted that the roughness in the topography images is different: KPFM uses tapping mode, which is very soft and hence more sensitive to the surface. That is why the topography image shows surface roughness (Fig. 3a). CP-AFM uses contact mode which is intrusive on the surface and may smooth out the surface roughness (see Fig. 3b). This was also reported by Li et al. [8]. Using a strong force on the tip is necessary to achieve a good contact. On the other hand, the CP-AFM mode degrades the material and the tip. The advantage is that CP-AFM is less sensitive to the contamination of the sample surface than KPFM.

The effect of the illumination is visible from the 3-μm-wide scans of the epitaxial silicon layer in both photocurrent and photovoltage images (Fig. 3c–f). The wafer does not show any difference with and without illumination because it is highly doped and grounded. Both a-Si:H and ITO layers are not visible on these images due to their very small thickness compared to the scan size and also possibly because they might have been degraded or removed during the polishing step of the cross section (rounding effect).

Some areas in photocurrent images acquired from measurements without LED illumination show photocurrent values above the lowest measurable level (10^−12^ A). This is due to the fact that AFM laser diode spills over the cantilever and creates a non-desired photocurrent. This phenomenon is amplified on rough areas where the cantilever undergoes both a deflection and a torsion causing an increase of AFM laser-induced current. We measured the influence of the AFM laser on both setups by measuring the open circuit voltage when all the sources of illumination were turned off, except for the AFM laser. The photovoltage reaches 14 mV for CP-AFM setup and 51 mV for KPFM setup. The parasitic effect of the laser on photocurrent and photovoltage measurements has also been identified and discussed by Feijfar et al. [5] and Zhang et al. [10].

On the photocurrent images, the imprint of the topography is quite evident: on the trenches (caused by the polishing) and on the edge of the sample, the current is much higher. This is due to an increase of the effective contact area between the probe and the sample. Therefore, more current can pass through the circuit. To achieve a more homogeneous image, the polishing process has to be improved. Focused ion beam (FIB) polishing, for instance, could enable smoother surfaces. KPFM measurements also show an imprint of topography on the surface potential images, especially on the edge of the sample. This topography cross talk was investigated by Barbet et al. [20]. However, the influence of topography on the measured values is smaller for KPFM than CP-AFM because KPFM leads to averaging of the signal on a wider area which screens local topographical influences.

### Influence of Increasing Illumination on KPFM and CP-AFM Profiles

**Fig. 4:**
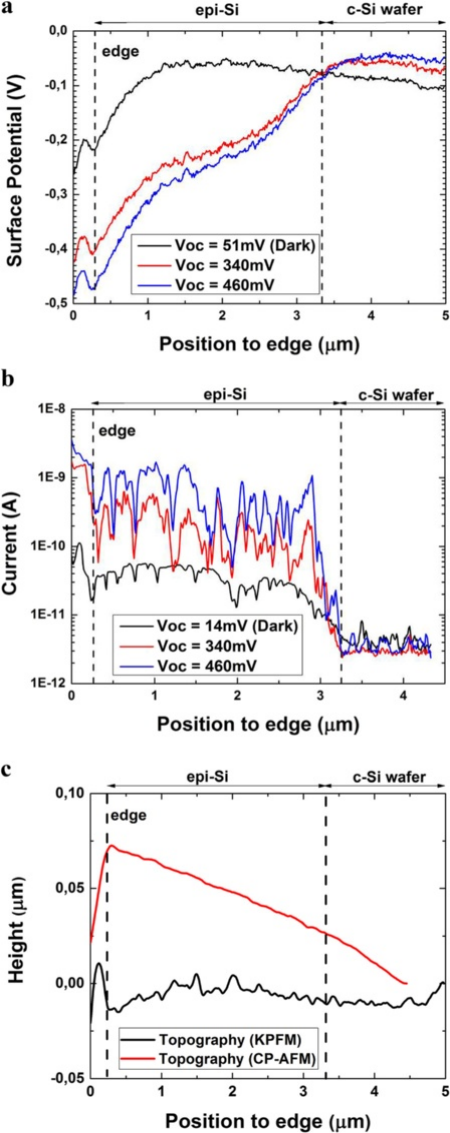
Photovoltage (**a**) and photocurrent (**b**) profiles measured at the sample cross section without LED illumination (*dark*) and under two intensities of illumination leading to *V*_oc_ = 340 mV and *V*_oc_ = 460 mV. Topography profiles associated to KPFM and CP-AFM (**c**)

Figure 4 shows KPFM and CP-AFM profiles along the PIN heterojunction without LED illumination and under two illumination intensities. The topography profiles obtained by both techniques allowing to position the edge of the sample are shown in Fig. 4c. As the *V*_oc_ increases logarithmically with the illumination intensity and it is easy to monitor, we present the profiles obtained under two levels of illumination resulting in *V*_oc_ of 340 and 460 mV. To reduce the noise in photovoltage measurement, we obtained a profile by averaging on 128 consecutive horizontal scan lines (which corresponds to 1.2 μm total vertical displacement). For photocurrent measurements, we took advantage of the signal increase inside the scratches and averaged on 13 horizontal scan lines which correspond to 120 nm. Successive AFM images have a tendency to drift. Therefore, profiles were drawn on easily identifiable areas of the photocurrent and photovoltage images. Then, they were reported as shown in Fig. 4.

The profiles show coherent behaviors: when the illumination intensity increases, the current increases in the intrinsic epi-Si layer (Fig. 4b) and the surface potential decreases by a difference correlated to the measured *V*_oc_ (Fig. 4a). Photovoltage profiles show a small noise related to thermal fluctuations of the cantilever; however this noise has been reduced by averaging. Contrary to what is expected theoretically, the junction at the interface between the crystalline silicon wafer and the epitaxial layer is not visible on the surface potential profile in the dark (Fig. 4a). Surface states leading to band bending complicate the analysis of KPFM measurement on pn junctions under open circuit conditions in the dark, as also reported by Kikukawa et al. [21]. This interpretation is confirmed in Fig. 3a which shows nanograins at the top of the cross sections. These nanograins can mask the potential contrast at the pn junction. The band bending is also the most likely reason for the surface potentials measured at the edge being only correlated and not equal to the *V*_oc_ of the solar cell. The difference in the surface potential on the wafer side between dark condition and under illumination is not expected and may result from a degradation phenomenon such as local oxidation or tip abrasion.

Photocurrent profiles (Fig. 4b) allow to precisely locate the c-Si/epi-layer interface. However, they show noise related to the topography, as discussed previously. When the probe is close to the edge of the cell, the current suddenly increases because the side of the tip touches the edge and hence the effective surface of contact is larger.

In Fig. 4, both KPFM and CP-AFM measurements along the cell cross section show that the profiles with and without illumination diverge strongly at the epi-Si/wafer p^++^ interface. Further in the epitaxial layer, the divergence between the two profiles is much smaller, as the profiles remain at a constant distance close to the edge. This is not expected for a standard PIN junction where the distance between profiles with and without illumination should increase progressively all along the intrinsic layer. Therefore, from the cross analysis of KPFM and CP-AFM measurements, we might suppose undesired phenomena taking place during the deposition process and affecting the opto-electrical behavior of the device. The first hypothesis is that the epitaxial layer was unintentionally n-doped leading to a junction of PN^-^N type instead of PIN type. The second hypothesis is the presence of a defective layer at the interface between the epitaxial layer and the crystalline wafer. It should be noted that the defective layer could have important fluctuations along the cross section leading to important electrical behaviors inhomogeneity. Both hypotheses are consistent with secondary ion mass spectrometry measurements showing a strong increase of the impurity content at the interface and an oxygen concentration of around 10^19^ cm^−3^ in the epitaxial layer. Hence, CP-AFM and KPFM are powerful tools to investigate the incidence of undesired process phenomena on different areas of the cross section.

### Study of Measurements Reproducibility

**Fig. 5:**
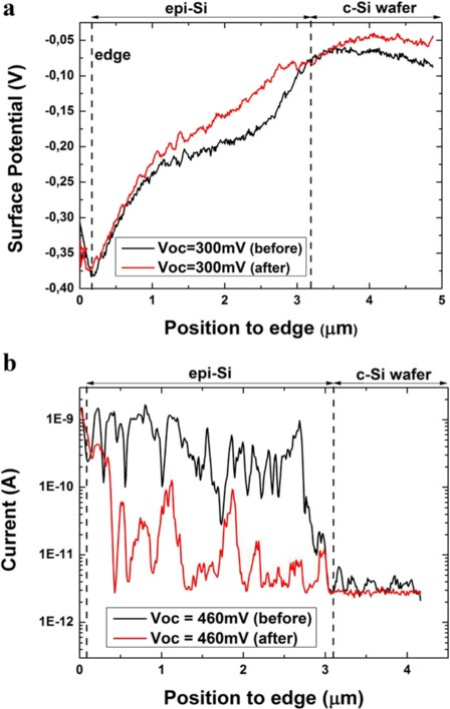
**a** Photovoltage profiles under an illumination corresponding to a *V*_oc_ of 300 mV before and after six scans. **b** Photocurrent measurements corresponding to a *V*_oc_ of 460 mV

We have also investigated the degradation of the photosignals. For that purpose, the profiles under the same illumination were performed before and after six scans under illumination for both discussed methods. Figure 5a shows that the photovoltage has already changed on the c-Si wafer side. This may result from a modification of the sample: tip-induced oxidation might happen despite the controlled nitrogen atmosphere. Also, we cannot exclude other effects related to the measurement itself, as degradation of the tip (its coating or in the tip radius). Moreover, these changes can also be attributed to surface effects induced by illumination such as local light-induced degradation or long-lived deep trap states, as was discussed by Zhang et al [10].

Figure 5b shows a strong degradation of photocurrent after six scans. This is particularly visible in the epitaxial silicon layer because the current signal in the wafer p^++^ region is below the detection level of the instrument so we can consider that it is equal to zero. The causes of degradation may again come from both the measurement process and/or a degradation of the sample. First, as above, there might be an abrasion of the tip coating and also a drift of the AFM laser on the photodetector during measurements. In fact, this can affect sensibly the force applied by the tip on the surface because a cantilever with strong spring constant (48 N/m) was used. Secondly, the degradation of the sample surface can come both from the indentation phenomena which was shown in Fig. 1 and from tip-induced oxidation during the scans. This phenomenon has been discussed by Vetushka et al. [22]. It should be noted that close to the edge of the sample, the values of current seem to be repeatable. This is due to two reasons: first, the area of contact corresponds to the flank of the tip which is larger and probably less degraded during the scans. Second, this region locally undergoes much less pressure than the flat region. Therefore, it is less degraded and the tip-induced oxidation is probably lower.

## Conclusions

In this paper, we have studied photovoltage and photocurrent with nanometer scale resolution on the cross section of an epitaxial silicon solar cell, using respectively Kelvin probe force microscopy and conducting probe atomic force microscopy. Using the area indented by CP-AFM as a geolocalization mark, we could perform measurements within a 100-μm distance, thus ensuring that the conditions of illumination and the local electrical properties of the solar cell are similar. By adapting our CP-AFM setup, we have proposed a new way of performing photocurrent measurements without an external voltage source, which enables an easier interpretation of the date. Photocurrent and photovoltage measurements are coherent with what is expected: photocurrent increases and surface potential decreases in the epitaxial silicon layer with increasing illumination intensity. The difficulties to achieve quantitative measurements are discussed. In particular, we highlight different sources of measurement artifacts. CP-AFM is very sensitive to the surface topography while KPFM is very sensitive to the surface condition (e.g., surface oxidation or surface contamination). The sample preparation steps (cleavage, polishing and cleaning) are therefore crucial to achieve accurate measurements. We also found that reproducibility of CP-AFM is more challenging than for KPFM measurements. The potential reasons are the degradation of both the area of the sample scanned and that of the measurement setup (abrasion of the tip and drift of the AFM laser). Despite these limitations, the comparison of measurements highlighted a dissymmetry on profiles along the PIN junction enabling to raise a hypothesis on undesired phenomena during the deposition process: unintentional n-doping of the epitaxial silicon layer and defective interface between the wafer and the epitaxial layer. Both these phenomena may have important consequences on the optoelectrical properties of the solar cell.

This study shows that scanning probe microscopy techniques are useful tools to analyze the homogeneity of PN junctions and the effect of illumination at the nanoscale. Ongoing work is carried out to investigate the homogeneity of electric field along the epitaxial silicon solar cell. We also expect that cross analysis using various nanoscale resolution characterization techniques on the same area of solar cells will enable a better understanding of the results provided by these techniques as well as a better understanding of the solar cells. Eventually, it will lead to the use of the full potentiality of scanning probe microscopy techniques for solar cell investigation.

## Abbreviations

AFM: atomic force microscopy
AM: amplitude modulation
a-Si:H: hydrogenated amorphous silicon
CIGS: CuIn_*x*_Ga_(1 − *x*)_Se_2_
CP-AFM: conducting probe atomic force microscopy
epi-PECVD: epitaxial layer deposited by plasma-enhanced chemical vapor deposition technique
FIB: focused ion beam
ITO: indium tin oxide
*I*-*V*: current voltage
KPFM: Kelvin probe force microscopy
LED: light-emitting diode
LPICM: Laboratoire d’Interfaces et Couches Minces
PECVD: plasma-enhanced chemical vapor deposition
Pt/Ir: platinium iridium
*R*_b_: barrier resistance
*R*_c_: contact resistance
RF-PECVD: radio frequency plasma-enhanced chemical vapor deposition
*R*_g_: geometrical resistance
*V*_oc_: open circuit voltage
WF: work function
